# Global prevalence of post-COVID-19 condition (Long COVID): a systematic review and meta-analysis of observational studies

**DOI:** 10.3389/fpubh.2026.1839646

**Published:** 2026-06-25

**Authors:** Samal Kassymbek, Aigul Abduldayeva, Nikolay Safonov

**Affiliations:** Department of Research Institute of Preventive Medicine named after Academician E. Dalenov, Astana Medical University, Astana, Kazakhstan

**Keywords:** heterogeneity, Long COVID, meta-analysis, post-acute sequelae of SARS-CoV-2 infection, post-COVID-19 condition, prevalence, PRISMA, systematic review

## Abstract

**Background:**

Long COVID is an umbrella term for persistent, relapsing, or newly developed health problems after SARS-CoV-2 infection, whereas post-COVID-19 condition (PCC) refers more specifically to the World Health Organization clinical case definition. Reported prevalence varies substantially because of differences in terminology, operational definitions, study populations, follow-up duration, hospitalization status, and symptom ascertainment. This systematic review and meta-analysis synthesized global observational evidence on Long COVID/PCC prevalence and examined major sources of variability.

**Methods:**

A systematic review and meta-analysis was conducted according to PRISMA 2020 principles. PubMed/MEDLINE, Scopus, Web of Science, and the WHO COVID-19 Global Literature Database were searched for studies published from 1 January 2020 to 23 February 2026. Observational studies were eligible if they included individuals with confirmed or probable SARS-CoV-2 infection, reported Long COVID/PCC or persistent post-COVID symptoms at least 4 weeks after acute infection, and provided numerator and denominator data. Terminology and operational case definitions were extracted separately. Prevalence estimates were pooled using a random-effects model with logit transformation and back-transformation. Heterogeneity was assessed using Cochran's Q, I^2^, tau^2^, and prediction intervals. Subgroup and sensitivity analyses were performed.

**Results:**

Twenty-two studies contributing 27 prevalence estimates and more than 200,000 participants were included. The primary estimate-level pooled prevalence was 30.8% (95% CI 26.8–35.0). Heterogeneity was extreme: Q = 8031.9, df = 26, *p* < 0.001; I^2^ = 99.7%; tau^2^ = 0.252 on the logit scale. The prediction interval was 14.0–54.8%. Pooled prevalence was higher among hospitalized cohorts (37.9%, 95% CI 29.5–47.1) than among non-hospitalized, community-based, or mixed cohorts (26.2%, 95% CI 22.0–30.9). WHO-defined PCC yielded lower prevalence (22.8%, 95% CI 14.3–34.4) than broader symptom-based definitions (39.7%, 95% CI 30.5–49.5). Cohort-level sensitivity analysis yielded a similar prevalence of 31.0% (95% CI 26.8–35.4).

**Conclusion:**

Long COVID/PCC represents a substantial post-acute public health burden. However, because heterogeneity was extreme, the pooled prevalence should be interpreted as a descriptive summary of heterogeneous observational evidence rather than as a single precise global rate. Standardized definitions, harmonized surveillance, transparent reporting, rehabilitation pathways, and multidisciplinary care models are needed.

## Introduction

The coronavirus disease 2019 (COVID-19) pandemic has generated a substantial long-term health burden beyond the acute phase of SARS-CoV-2 infection. A proportion of infected individuals experience persistent, relapsing, or newly developed symptoms that may affect daily functioning, work capacity, quality of life, and demand for healthcare services. These post-acute consequences have been described using several terms, including Long COVID, post-COVID-19 condition, post-COVID syndrome, post-acute sequelae of SARS-CoV-2 infection (PASC), and persistent post-COVID symptoms (1–6).

Terminology in this field has evolved throughout the pandemic and remains incompletely standardized. In this review, Long COVID is used as an umbrella term for post-acute health consequences attributed to SARS-CoV-2 infection. Post-COVID-19 condition (PCC) refers more specifically to the World Health Organization clinical case definition, which describes a condition occurring usually 3 months after the onset of COVID-19, with symptoms lasting at least 2 months, not explained by an alternative diagnosis, and generally affecting everyday functioning (1, 2). The WHO also describes PCC as a condition commonly referred to as Long COVID (3, 4). The Centers for Disease Control and Prevention uses Long COVID and Post-COVID Conditions as closely related terms, whereas the National Institute for Health and Care Excellence distinguishes ongoing symptomatic COVID-19 from 4 to 12 weeks and post-COVID-19 syndrome beyond 12 weeks within the broader framework of long-term effects of COVID-19 (5, 6).

Therefore, these terms should not be interpreted as strictly separate and mutually exclusive disease entities in the epidemiological literature. Rather, they represent overlapping labels applied to a broad post-acute construct, with the actual case definition depending on the time threshold, symptom list, requirement for functional impairment, exclusion of alternative diagnoses, attribution to SARS-CoV-2 infection, and method of symptom ascertainment. The post-COVID functional status scale and related functional assessment tools should also be distinguished from diagnostic labels: they measure functional impact after COVID-19 but do not themselves define a distinct disease entity. For this reason, the present review extracts both the terminology used by each primary study and the operational case definition applied for prevalence estimation. Meta-analytic subgrouping is based on operational criteria rather than terminology alone.

Reported prevalence estimates of Long COVID/PCC vary widely across studies. Earlier systematic reviews and large population-based studies have shown that the estimated frequency of persistent symptoms after SARS-CoV-2 infection depends strongly on study design, population, acute disease severity, follow-up duration, case definition, and symptom assessment method (7–10). Some studies use broad definitions such as any persistent symptom after four weeks, whereas others require symptoms lasting at least 12 weeks, WHO-defined PCC, functional impairment, or exclusion of alternative diagnoses. Studies applying different definitions to the same population have demonstrated that prevalence estimates can change substantially depending on the operational definition used (11, 12).

This variability has direct implications for epidemiological interpretation. A pooled prevalence estimate can be useful for summarizing the published observational literature, but it should not be interpreted without attention to heterogeneity. In prevalence research on Long COVID, heterogeneity is not only a statistical challenge but also a substantive finding: it reflects the current lack of harmonization in terminology, diagnostic thresholds, follow-up windows, study populations, and measurement strategies. Consequently, the main value of a global synthesis is not merely to produce a single pooled estimate, but to quantify the range of reported prevalence and identify the methodological factors that shape these estimates.

Several population and clinical factors may influence reported prevalence. Hospitalized patients and individuals with more severe acute COVID-19 generally show higher frequencies of persistent symptoms than non-hospitalized or community-based populations (13–16). Pediatric and adult populations may also differ in symptom patterns, reporting, and functional consequences. In addition, many studies rely on self-reported questionnaires or telephone interviews, which can capture patient experience but may introduce recall bias, reporting bias, and difficulty distinguishing SARS-CoV-2-attributable symptoms from background symptoms or comorbid conditions. Studies with baseline symptom data or control groups may therefore yield different estimates from studies based only on post-infection symptom reporting (12, 17).

The pandemic context may further contribute to variability. Studies included in the Long COVID literature span different phases of the pandemic, including pre-vaccination and vaccination periods, different SARS-CoV-2 variant waves, changes in testing access, and evolving clinical awareness of Long COVID. Vaccination status, reinfection history, variant period, and healthcare access are not consistently reported across studies, but these factors may influence both the risk of persistent symptoms and the likelihood that symptoms are detected, reported, or clinically coded.

From a public health perspective, Long COVID/PCC represents an important challenge for healthcare systems even if its exact prevalence remains uncertain. Persistent post-COVID symptoms may increase demand for primary care, rehabilitation, respiratory and cardiovascular assessment, cognitive and mental health support, and multidisciplinary care pathways. For surveillance and planning, health systems need not only pooled estimates but also standardized definitions, harmonized follow-up protocols, transparent reporting of operational criteria, and stratified estimates by population type and severity.

Given the rapidly expanding and methodologically heterogeneous literature, an updated systematic review and meta-analysis is needed to synthesize global observational evidence on the prevalence of Long COVID/PCC. The objective of this review was to estimate the pooled prevalence of Long COVID/PCC in observational studies and to examine how prevalence estimates vary according to operational case definition, hospitalization status, geographic region, age structure, and follow-up duration. The primary outcome was the prevalence of Long COVID/PCC or persistent post-COVID symptoms as defined by the original studies, with WHO-defined PCC examined separately as a predefined subgroup.

## Methods

### Study design and reporting framework

This systematic review and meta-analysis was conducted in accordance with the Preferred Reporting Items for Systematic Reviews and Meta-Analyses 2020 statement (18). The literature search and reporting of search methods were additionally structured to improve reproducibility in line with PRISMA-S recommendations, including explicit reporting of information sources, search dates, search terms, and database-specific search strategies (19).

### Research question and eligibility framework

The review question was structured using an adapted Population-Exposure-Outcome framework. The population of interest included individuals with confirmed or probable SARS-CoV-2 infection. The exposure was the post-acute period following COVID-19. The outcome was the prevalence of Long COVID, post-COVID-19 condition, post-acute sequelae of SARS-CoV-2 infection, post-COVID syndrome, or persistent post-COVID symptoms as defined by the original studies. Eligible study designs included observational cohort studies, cross-sectional studies, registry-based studies, and population-based surveys reporting primary prevalence data.

### Search strategy

A systematic literature search was conducted in PubMed/MEDLINE, Scopus, Web of Science, and the WHO COVID-19 Global Literature Database. The search covered studies published from 1 January 2020 to 23 February 2026. The final search was conducted on 23 February 2026. Reference lists of relevant reviews and all included studies were additionally screened manually to identify eligible studies not captured by database searching.

The search strategy combined controlled vocabulary terms, where available, and free-text terms related to SARS-CoV-2 infection, Long COVID/post-COVID-19 condition, persistent symptoms, epidemiology, and prevalence. No restrictions were applied by geographic region at the search stage. Studies published in English or Russian were eligible for inclusion. The language restriction is acknowledged as a potential source of language bias. Full database-specific search strategies are provided in Supplementary Table S1.

### Eligibility criteria

Studies were eligible for inclusion if they met the following criteria: included individuals with confirmed or probable SARS-CoV-2 infection; reported the prevalence, frequency, cumulative incidence, or proportion of Long COVID, post-COVID-19 condition, PASC, post-COVID syndrome, or persistent post-COVID symptoms; provided sufficient numerator and denominator data to calculate a prevalence estimate, or reported a prevalence estimate with a clearly defined denominator; assessed outcomes at least 4 weeks after acute SARS-CoV-2 infection; used an observational design, including cohort, cross-sectional, registry-based, or population survey designs; and reported original primary data.

The minimum follow-up threshold of 4 weeks was used because early Long COVID studies and several national or operational definitions classified persistent symptoms from 4 weeks after acute infection as post-acute or Long COVID-related manifestations. However, this threshold is broader than the WHO clinical case definition of PCC, which requires symptoms usually occurring 3 months from COVID-19 onset and lasting for at least 2 months, without an alternative diagnosis (1, 2). Therefore, studies using broader four-week or symptom-based definitions were retained for the overall descriptive synthesis, while studies applying the WHO definition or a WHO-compatible operationalization were analyzed separately in subgroup analyses.

Studies were excluded if they were case reports, small case series, narrative reviews, editorials, commentaries, or letters without original prevalence data; modeling or simulation studies without primary observational prevalence data; studies without denominator data; studies focused exclusively on single-organ complications without reporting an overall Long COVID/PCC or persistent symptom prevalence estimate; studies assessing only acute COVID-19 outcomes without post-acute follow-up; or duplicate reports from another included study without a distinct population, follow-up time point, subgroup, or definition.

For the purposes of this review, an overall Long COVID or PCC prevalence estimate was defined as a study-level estimate of the proportion of individuals meeting the authors' overall Long COVID/PCC case definition or reporting at least one persistent symptom across any organ system after acute SARS-CoV-2 infection. Studies limited exclusively to one organ domain, such as only pulmonary fibrosis, myocarditis, renal impairment, or neurocognitive impairment, were excluded unless they also reported an overall syndrome-level Long COVID/PCC prevalence estimate.

### Study selection

All retrieved records were imported into reference management software, and duplicates were removed before screening. Two reviewers independently screened titles and abstracts against the eligibility criteria. Potentially eligible studies underwent full-text assessment. Disagreements were resolved through discussion and consensus; when consensus could not be reached, a third reviewer was consulted. Inter-rater agreement was not formally quantified.

### Data extraction

Data were independently extracted by two reviewers using a standardized extraction form developed before quantitative synthesis. Extracted variables included author and year of publication, country, geographic region, study design, data source, recruitment setting, sample size, age group, hospitalization status, follow-up duration, Long COVID/PCC definition, symptom assessment method, number of Long COVID cases, denominator, prevalence estimate, and risk-of-bias category.

When the original publication reported prevalence as a percentage without an explicit numerator, the numerator was reconstructed from the reported sample size and percentage and rounded to the nearest integer. Reconstructed numerators were flagged in the extraction table. Definitions of Long COVID were extracted as reported by the original authors and then categorized for analysis into operational groups: WHO-defined PCC, persistent symptoms lasting at least 12 weeks, broader post-acute symptom definitions beginning at 4 weeks or later, and other study-specific definitions. Follow-up duration was extracted as reported and, where possible, converted into months to improve comparability across studies.

For each included study, we extracted both the label used by the authors, such as Long COVID, PCC, post-COVID syndrome, PASC, persistent post-COVID symptoms, or post-acute sequelae of SARS-CoV-2 infection, and the operational case definition applied in the analysis. Because terminology did not consistently map onto a single diagnostic algorithm across studies, meta-analytic subgroups were defined according to operational criteria rather than terminology alone.

### Handling of multiple estimates from the same study

Some studies reported more than one eligible prevalence estimate because they presented different follow-up time points, age groups, hospitalization strata, or case definitions. For transparency, all eligible estimates were extracted and presented in the evidence table. These estimates were retained in the primary estimate-level synthesis when they represented clinically or methodologically distinct strata, such as adult vs. pediatric participants, hospitalized vs. non-hospitalized participants, different follow-up time points, or different operational definitions of Long COVID.

We recognized that including multiple estimates from the same publication or cohort may introduce statistical non-independence. Therefore, the primary analysis was interpreted as an estimate-level descriptive synthesis rather than as a model assuming that all estimates were fully independent study effects. To assess the potential influence of non-independence, we conducted a cohort-level sensitivity analysis including only one estimate per cohort or clinically distinct stratum. For this sensitivity analysis, estimates were selected according to a predefined hierarchy: first, estimates most closely aligned with the WHO PCC definition; second, estimates based on symptoms persisting at least 12 weeks; third, the longest available follow-up time point; and fourth, the broadest or most representative population estimate when several subgroup estimates were available.

### Quality assessment

The methodological quality of included studies was evaluated using the Newcastle-Ottawa Scale (NOS) adapted for observational studies and prevalence research (20). The assessment considered participant selection, comparability or representativeness, outcome definition and assessment, and follow-up or response completeness. Studies were classified as low, moderate, moderate-high, or high risk of bias. Quality assessment was performed independently by two reviewers. Disagreements were resolved by consensus. The limitations of NOS reproducibility and reviewer variation were considered when interpreting quality categories (21). Risk-of-bias results are provided in Supplementary Table S3.

### Statistical analysis

The primary outcome was the prevalence of Long COVID/PCC, calculated as the number of participants meeting the study-specific Long COVID/PCC definition divided by the total number of participants assessed at follow-up. Because raw proportions may have unstable variances, all prevalence estimates were transformed using the logit transformation before pooling. Pooled estimates and 95% confidence intervals were then back-transformed to the proportion scale for interpretation.

The primary meta-analysis was performed using a random-effects model. The DerSimonian-Laird estimator was used as the primary estimator of between-study variance to maintain comparability with conventional prevalence meta-analyses (22). Given the expected clinical and methodological heterogeneity across studies, pooled estimates were interpreted descriptively rather than as a single true global prevalence.

Statistical heterogeneity was assessed using Cochran's Q statistic, the corresponding *p*-value, the I^2^ statistic, and the between-study variance tau^2^ (23). Tau^2^ was estimated on the logit scale. Prediction intervals were calculated to indicate the expected range of prevalence estimates in a future comparable study. No zero-event prevalence estimates were identified; therefore, no continuity correction was required.

Prespecified subgroup analyses were conducted according to geographic region, hospitalization status, diagnostic definition, age group, and follow-up duration where data permitted. Differences between subgroups were assessed using the Q test for subgroup differences on the logit-transformed scale. These subgroup tests were interpreted as exploratory because of extreme within-subgroup heterogeneity and the small number of estimates in some subgroups.

Sensitivity analyses included leave-one-out analysis, cohort-level sensitivity analysis including only one estimate per cohort or clinically distinct stratum, and alternative interpretation of pooled estimates with emphasis on prediction intervals rather than confidence intervals alone. Potential small-study effects and publication bias were assessed using funnel plot visualization and Egger's regression test (24). Because funnel plots and Egger's test have limited interpretability in prevalence meta-analyses with substantial heterogeneity, these analyses were interpreted cautiously and were not considered definitive evidence for or against publication bias. All statistical analyses were performed using R statistical software with validated meta-analysis packages (25, 26). The double-arcsine transformation was not used as the primary approach because of known interpretability and back-transformation limitations in meta-analysis of single proportions (27).

## Results

### Study selection

**Figure 1:**
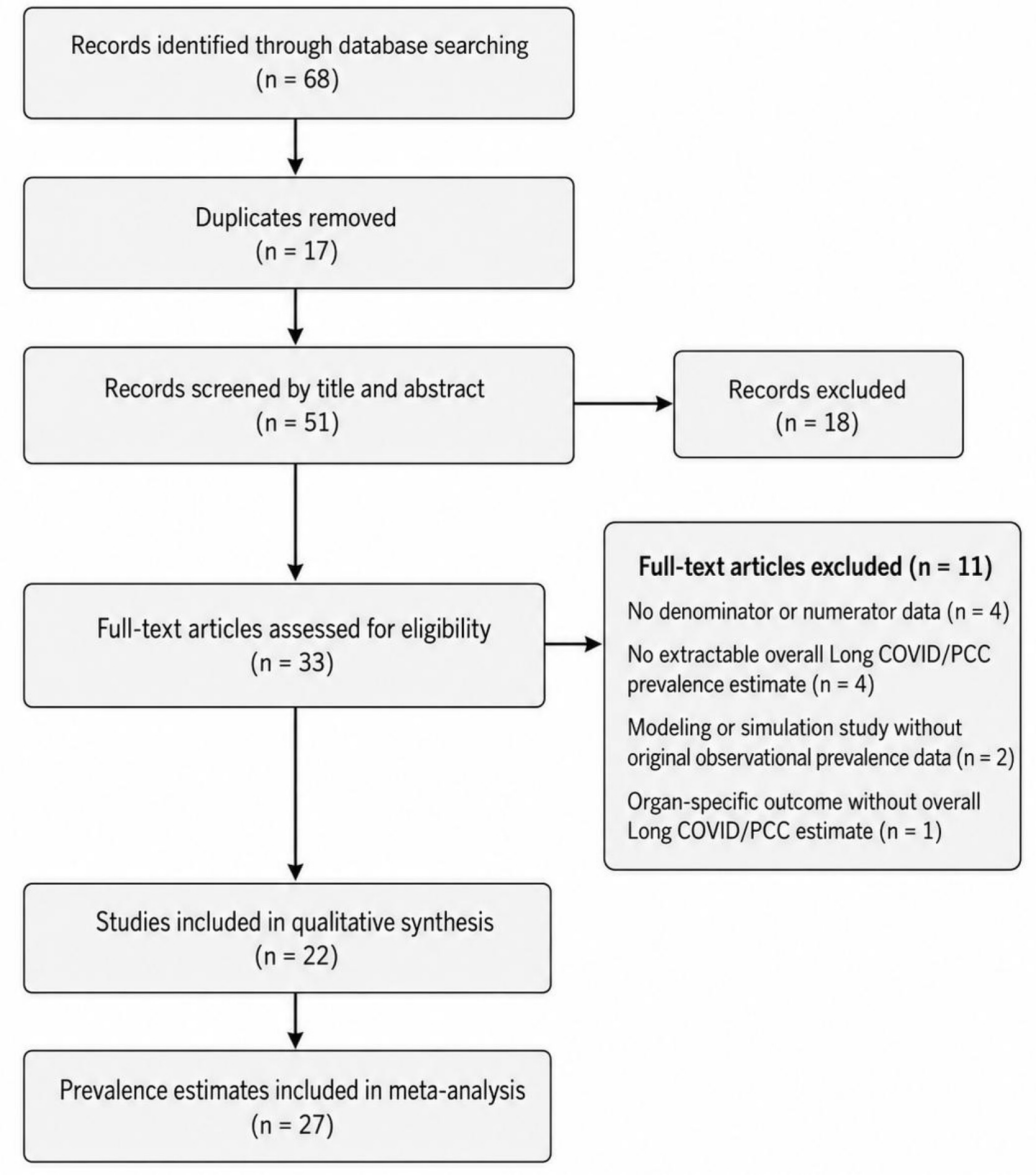
PRISMA 2020 flow diagram of study selection. PCC, post-COVID-19 condition.

The database search identified 68 records. After removal of 17 duplicates, 51 records were screened by title and abstract. Of these, 18 records were excluded because they were not relevant to the review question. The remaining 33 full-text articles were assessed for eligibility. After full-text review, 11 articles were excluded for the following reasons: absence of denominator or numerator data (*n* = 4), no extractable overall Long COVID/PCC prevalence estimate (*n* = 4), modeling or simulation study without original observational prevalence data (*n* = 2), and organ-specific outcome without an overall Long COVID/PCC estimate (*n* = 1). Finally, 22 studies met the inclusion criteria and were included in the systematic review. Several studies contributed more than one prevalence estimate because they reported different follow-up periods, age groups, hospitalization strata, or operational definitions. In total, 27 prevalence estimates were included in the primary estimate-level meta-analysis (Figure 1).

### Study characteristics

The 22 included studies were conducted across Europe, Asia, Africa, South America, and the Middle East. Sample sizes ranged from 315 participants in smaller clinical cohorts to more than 92,000 participants in national population-based surveys. Study populations included hospitalized patients, non-hospitalized or community-based participants, mixed-severity cohorts, and pediatric cohorts. Follow-up duration ranged from approximately 3 months to 4 years after acute SARS-CoV-2 infection. Definitions of Long COVID varied substantially across studies. Some studies applied the WHO PCC definition, whereas others used persistent symptoms lasting at least 12 weeks, broader symptom-based definitions at fixed follow-up time points, functional case definitions, or self-reported non-recovery. Table 1 summarizes the main characteristics of the included studies, while Supplementary Table S2 provides estimate-level extraction details, including cohort ID, estimate ID, operational definition, follow-up duration, and inclusion in the cohort-level sensitivity analysis. The included studies and prevalence estimates were based on observational evidence from the studies listed in Table 1 and Supplementary Table S2 (29–47).

**Table 1:** Overview of included evidence by region and population setting.

Region	Evidence block	Studies/ estimates	Population/ setting	Operational definitions	Follow-up	Prevalence range	Risk of bias
Europe	Population/ community studies	6 studies/ 7 estimates	Adults; mostly non-hospitalized or mixed community/ primary-care samples	WHO PCC; ≥12-week symptoms; broad/ fixed symptoms; functional definition	3–12 mo	8.0%−49.3%	Low-moderate to moderate-high
Europe	Hospitalized/ clinical follow-up cohorts	3 studies/ 5 estimates	Adults; hospitalized or clinically followed strata	WHO PCC; broad/ fixed symptom definitions	6–24 mo	34.0%−67.5%	Moderate-high
Europe	Pediatric hospitalized cohort	1 study/ 2 estimates	Children after hospitalization	WHO-defined PCC	6 and 12 mo	11.1%−20.0%	Moderate-high
Asia	Community or mostly non-hospitalized studies	5 studies/ 5 estimates	Adults; community, household, registry/ survey, or mostly mild cases	WHO PCC; ≥12-week symptoms	3 mo-4 y	11.8%−43.2%	Low-moderate to moderate-high
Asia	Hospitalized cohorts	4 studies/ 4 estimates	Adults after hospitalization	≥12-week symptoms; broad/ fixed symptom definitions	12–36 mo	11.4%−54.6%	Moderate-high
Africa	Mixed-severity and hospitalized/ non-hospitalized strata	2 studies/ 3 estimates	Adults; hospitalized, non-hospitalized, and mixed cohorts	≥12-week symptoms; broad/ fixed symptom definitions	3–6 mo	18.5%−46.7%	Moderate-high
South America	Mixed-severity cohort	1 study/ 1 estimate	Adults/ older adults; ambulatory, ward, and ICU history	Broad/ fixed symptom definition	12 mo	64.2%	Moderate

### Terminology and operational definitions

Terminology and operational definitions were not fully aligned across included studies. The same broad post-COVID construct was described using different labels, including Long COVID, PCC, post-COVID syndrome, persistent symptoms, and PASC. Conversely, studies using similar terminology often applied different operational criteria, including WHO-defined PCC, symptoms lasting at least 12 weeks, symptom-any definitions at fixed follow-up points, functional case definitions, or self-reported non-recovery. Therefore, prevalence estimates were categorized according to operational case definitions rather than terminology alone. This approach was used to distinguish studies applying WHO-defined PCC from those using broader symptom-based or study-specific definition.

Compact main-manuscript version. Full estimate-level extraction data are provided in Supplementary Table S2.

### Global prevalence of Long COVID

**Figure 2:**
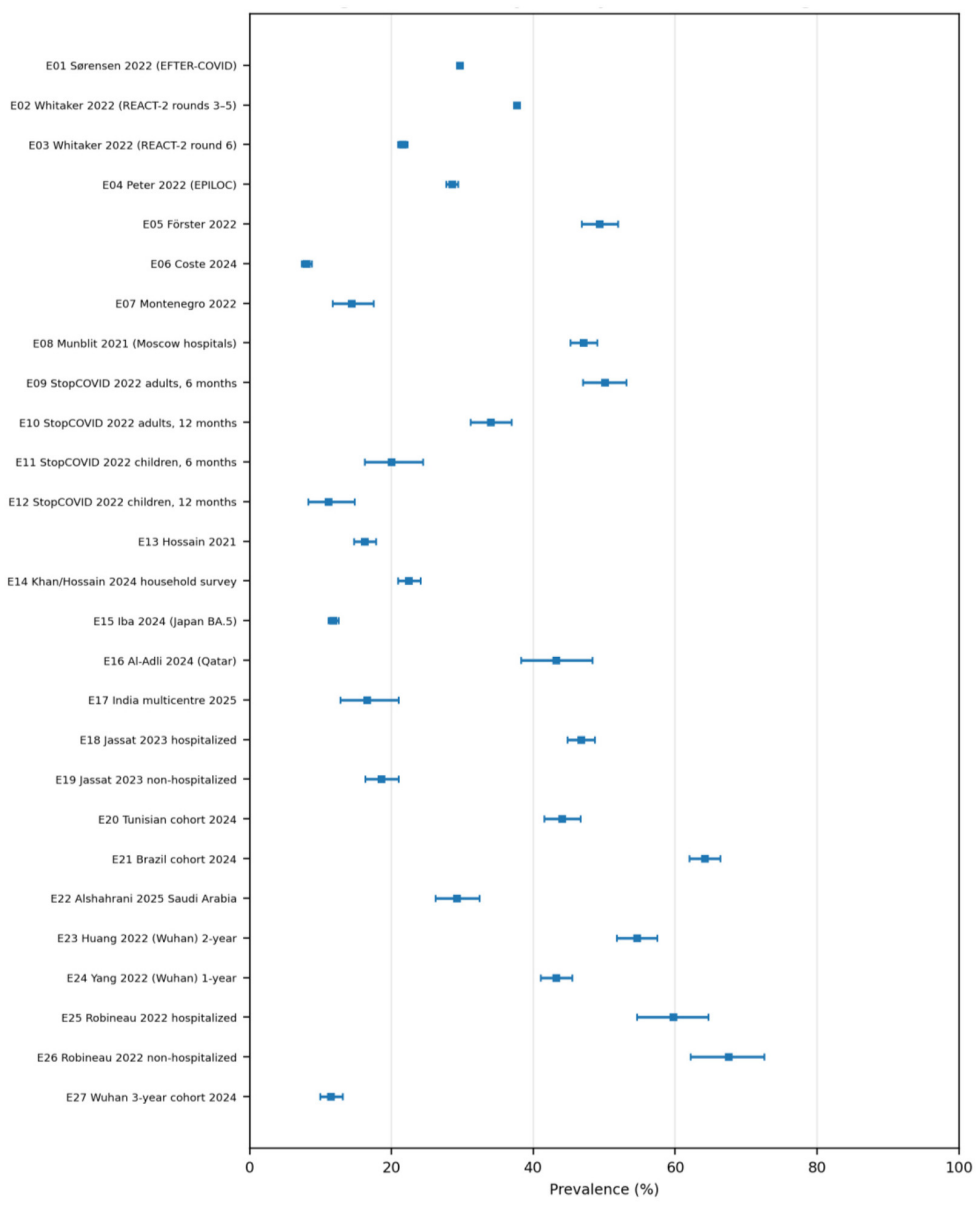
Forest plot of the pooled prevalence of long COVID across included estimates. The forest plot displays 27 prevalence estimates from 22 observational studies. Individual estimates were pooled using a random-effects model with logit transformation and back-transformation to the proportion scale. The pooled prevalence was 30.8% (95% CI 26.8–35.0). Heterogeneity was extreme (Q = 8,031.9, df = 26, *p* < 0.001; I^2^ = 99.7%; tau^2^ = 0.252 on the logit scale). The prediction interval ranged from 14.0 to 54.8%.

The pooled prevalence of Long COVID across 27 prevalence estimates was calculated using a random-effects model with logit transformation and back-transformation to the proportion scale (Figure 2). The overall pooled prevalence was 30.8% (95% CI 26.8–35.0). Statistical heterogeneity was extreme: Cochran's Q = 8,031.9, df = 26, *p* < 0.001; I^2^ = 99.7%. The estimated between-study variance was tau^2^ = 0.252 on the logit scale. The prediction interval ranged from 14.0 to 54.8%, indicating that prevalence estimates in future comparable studies may vary widely across settings, populations, and operational definitions. Because of this extreme heterogeneity, the pooled estimate should be interpreted as a broad descriptive summary of the published observational evidence rather than as a single precise global prevalence.

### Subgroup analyses

Subgroup analysis by hospitalization status showed a higher pooled prevalence among hospitalized cohorts than among non-hospitalized, community-based, or mixed cohorts. In the primary estimate-level analysis, hospitalized cohorts had a pooled prevalence of 37.9% (95% CI 29.5–47.1; k = 11), whereas non-hospitalized/community/mixed cohorts had a pooled prevalence of 26.2% (95% CI 22.0–30.9; k = 16). The exploratory test for subgroup differences was statistically significant (Q-between = 987.5, df = 1, *p* < 0.001). However, heterogeneity remained high within both subgroups, indicating that hospitalization status alone did not fully explain between-study variability.

Prevalence estimates varied according to the operational definition of Long COVID/PCC. Studies applying WHO-defined PCC showed a pooled prevalence of 22.8% (95% CI 14.3–34.4; k = 9), while studies using 12-week persistent symptom definitions showed a pooled prevalence of 28.7% (95% CI 20.5–38.6; k = 6). Studies using broader symptom-based definitions assessed at fixed follow-up points yielded a higher pooled prevalence of 39.7% (95% CI 30.5–49.5; k = 11). One study-specific functional definition yielded a prevalence estimate of 28.5% (95% CI 27.7–29.3; k = 1). The exploratory test for subgroup differences was statistically significant (Q-between = 2,046.2, df = 3, *p* < 0.001).

Regional subgroup analyses showed substantial variability. In the primary estimate-level analysis, pooled prevalence was 31.6% in Europe (95% CI 27.0–36.5; k = 14), 25.2% in Asia (95% CI 15.9–37.5; k = 9), 35.1% in Africa (95% CI 21.6–51.6; k = 3), and 64.2% in South America (95% CI 61.9–66.3; k = 1). The exploratory Q test for subgroup differences was statistically significant (Q-between = 1,583.8, df = 3, *p* < 0.001). Regional comparisons should be interpreted cautiously because some regions were represented by few estimates, and South America by only one estimate.

Prevalence estimates also varied by follow-up duration. Studies with follow-up between 3 and < 6 months showed a pooled prevalence of 26.1% (95% CI 17.5–37.0; k = 7; I^2^ = 99.8%). Studies with follow-up between 6 and 12 months showed a pooled prevalence of 29.4% (95% CI 23.7–35.8; k = 15; I^2^ = 99.6%). Studies with follow-up beyond 12 months showed a pooled prevalence of 43.0% (95% CI 23.0–65.5; k = 5; I^2^ = 99.4%). The exploratory test for subgroup differences was statistically significant (Q-between = 524.9, df = 2, *p* < 0.001). These results should be interpreted cautiously because follow-up duration was closely related to study design, population type, hospitalization status, and operational definition.

### Sensitivity analyses

Leave-one-out sensitivity analysis showed that the pooled prevalence estimate remained stable after sequential exclusion of individual estimates. The pooled prevalence varied between 29.2 and 32.1%, indicating that no single estimate disproportionately influenced the overall result. To assess the potential influence of non-independence from multiple estimates within the same cohort, we conducted a cohort-level sensitivity analysis including only one estimate per cohort or clinically distinct stratum. In this analysis, 24 estimates were retained. The pooled prevalence remained highly consistent with the primary estimate-level analysis: 31.0% (95% CI 26.8–35.4), compared with 30.8% (95% CI 26.8–35.0) in the full estimate-level analysis. Heterogeneity remained extreme: Cochran's Q = 6,996.6, df = 23, *p* < 0.001; I^2^ = 99.7%; tau^2^ = 0.247 on the logit scale. The prediction interval ranged from 14.2 to 54.8%. These findings indicate that the overall interpretation was not materially driven by repeated estimates from the same cohort. Subgroup and sensitivity analyses are summarized in Table 2.

**Table 2:** Subgroup and sensitivity analyses of long COVID prevalence.

Subgroup	Category	k	Pooled prevalence %	95% CI	Prediction interval	I^2^ %	Q-between
Overall	All estimates	27	30.8	26.8–35.0	14.0–54.8	99.7	—
Hospitalization	Hospitalized	11	37.9	29.5–47.1	—	99.1	987.5, *p* < 0.001
Hospitalization	Non-hospitalized/community/mixed	16	26.2	22.0–30.9	—	99.7	—
Definition	WHO-defined PCC	9	22.8	14.3–34.4	—	99.5	2,046.2, *p* < 0.001
Definition	≥12-week persistent symptoms	6	28.7	20.5–38.6	—	99.7	—
Definition	Broad/fixed symptom definitions	11	39.7	30.5–49.5	—	99.6	—
Definition	Functional/study-specific	1	28.5	27.7–29.3	—	—	—
Region	Europe	14	31.6	27.0–36.5	16.7–51.5	99.7	1,583.8, *p* < 0.001
Region	Asia	9	25.2	15.9–37.5	5.2–67.5	99.6	—
Region	Africa	3	35.1	21.6–51.6	12.3–67.6	99.2	—
Region	South America	1	64.2	61.9–66.3	61.9–66.3	—	—
Follow-up	3- < 6 months	7	26.1	17.5–37.0	7.7–59.8	99.8	524.9, *p* < 0.001
Follow-up	6–12 months	15	29.4	23.7–35.8	11.5–57.1	99.6	—
Follow-up	>12 months	5	43.0	23.0–65.5	7.3–87.8	99.4	—
Sensitivity	One estimate per cohort/stratum	24	31.0	26.8–35.4	14.2–54.8	99.7	—

Subgroup and sensitivity analyses of long COVID prevalence. Pooled estimates were calculated using random-effects meta-analysis with logit transformation and back-transformation to the proportion scale. Q-between values represent exploratory tests for subgroup differences.

### Small-study effects and publication bias

**Figure 3:**
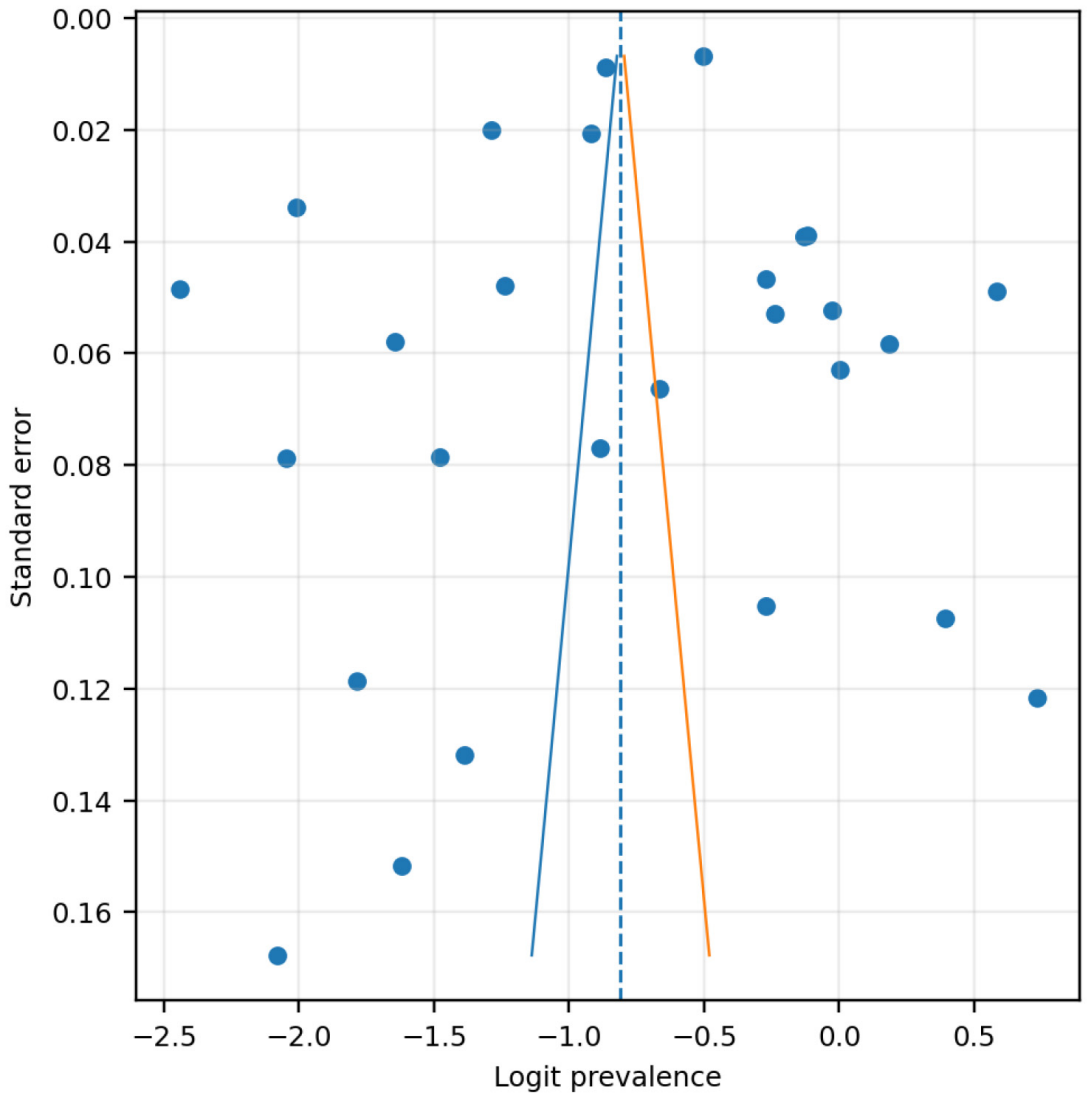
Funnel plot for visual assessment of small-study effects. Funnel plot visualization did not show clear asymmetry, and Egger's regression test was not statistically significant (intercept = −2.75, SE = 7.50, *p* = 0.72).

Funnel plot visualization and Egger's regression test were used to explore potential small-study effects (Figure 3). Visual inspection of the funnel plot did not show clear asymmetry. Egger's regression test was not statistically significant (intercept = −2.75, SE = 7.50, *p* = 0.72). However, this result should be interpreted cautiously. Funnel plot asymmetry tests have limited power and limited interpretability in prevalence meta-analyses with extreme heterogeneity. Therefore, the absence of statistically significant asymmetry should not be interpreted as definitive evidence that publication bias was absent.

## Discussion

### Principal findings

This systematic review and meta-analysis synthesized global observational evidence on the prevalence of Long COVID/PCC across 22 studies contributing 27 prevalence estimates. The primary estimate-level random-effects meta-analysis showed a pooled prevalence of 30.8% (95% CI 26.8–35.0). However, heterogeneity was extreme (I^2^ = 99.7%), and the prediction interval was wide (14.0%−54.8%), indicating that prevalence estimates in future comparable studies may vary substantially across settings, populations, and operational definitions.

Therefore, the pooled estimate should not be interpreted as a single precise global prevalence of Long COVID. Rather, it should be understood as a broad descriptive summary of heterogeneous observational evidence. The central finding of this review is that Long COVID prevalence estimates are highly context-dependent and vary according to operational case definition, follow-up duration, hospitalization status, age structure, symptom ascertainment method, and pandemic context.

Importantly, the cohort-level sensitivity analysis including only one estimate per cohort or clinically distinct stratum produced a highly consistent pooled estimate of 31.0% (95% CI 26.8–35.4). This suggests that the main result was not materially driven by repeated estimates from the same cohort. Instead, the high heterogeneity appears to reflect genuine methodological and clinical variability in the Long COVID literature.

### Interpretation of heterogeneity and justification for pooling

**Figure 4:**
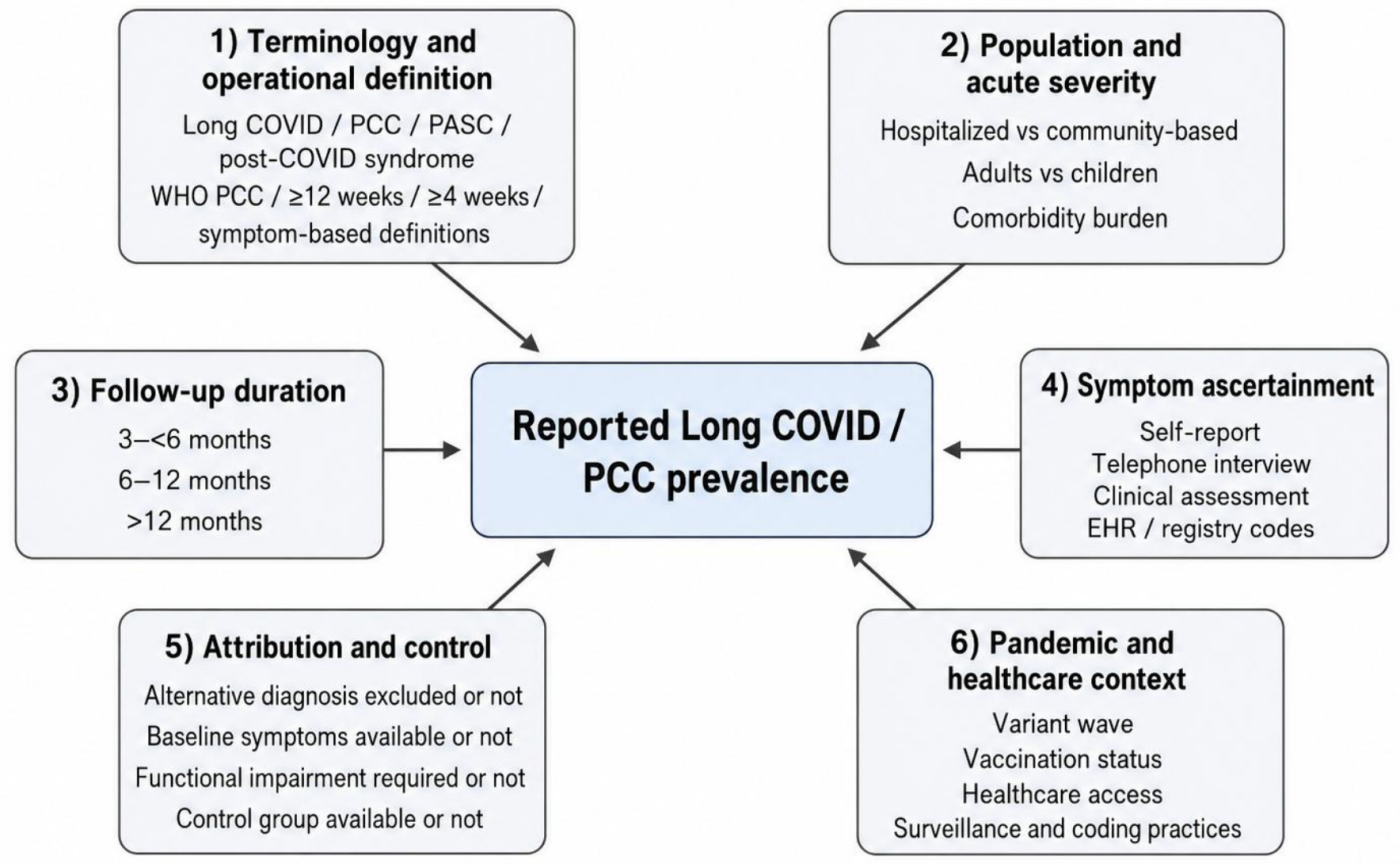
Main sources of heterogeneity in long COVID prevalence estimates. Reported prevalence estimates are influenced by terminology, operational case definition, population characteristics, hospitalization status, follow-up duration, method of symptom ascertainment, attribution rules, availability of baseline/control data, vaccination status, variant period, and healthcare-system context. This framework illustrates why the pooled estimate should be interpreted as a descriptive summary of heterogeneous observational evidence rather than a single true global prevalence.

The extremely high heterogeneity observed in this review is both a statistical limitation and a substantive finding (Figure 4). It limits the precision and generalizability of any single pooled prevalence estimate, but it also demonstrates a core problem in the epidemiology of Long COVID: studies do not measure the same construct in a uniform way. Similar conclusions have been shown in regional analyses where the overall pooled estimate was interpreted as an average across different definitions, follow-up durations, and populations rather than as a single true regional prevalence.

Quantitative pooling was retained because the objective of this review was not to claim a single underlying global prevalence, but to summarize the published observational evidence and quantify the extent of variability across studies. This approach is appropriate when the pooled estimate is interpreted descriptively, accompanied by prediction intervals, subgroup analyses, sensitivity analyses, and a cautious discussion of limitations.

In this context, the prediction interval is particularly important. While the confidence interval describes uncertainty around the average pooled estimate, the prediction interval reflects the expected range of prevalence estimates in future settings. The wide prediction interval in our analysis supports the conclusion that Long COVID prevalence should be interpreted as a range of context-specific estimates rather than as one universal global rate.

### Terminology and operational definitions

A major contributor to heterogeneity was the inconsistent relationship between terminology and operational case definitions. Long COVID, PCC, post-COVID syndrome, PASC, and related terms are often used in overlapping ways in the literature. However, they do not always correspond to a single diagnostic algorithm.

In this review, Long COVID was treated as an umbrella term for post-acute health consequences attributed to SARS-CoV-2 infection, whereas WHO-defined PCC was treated as a more specific clinical case definition. The reviewer's concern regarding terminology is therefore important. These labels should not be treated as perfectly interchangeable. At the same time, the primary epidemiological literature does not support a strict one-to-one interpretation in which each term represents a mutually exclusive disease entity. For this reason, our revised analysis extracted both the label used by the study authors and the operational definition applied in each study, and subgroup analyses were based on operational criteria rather than terminology alone.

This approach is supported by studies showing that prevalence can vary substantially when different definitions are applied to the same population. For example, national population-based data from France showed that prevalence estimates differed markedly depending on whether WHO, NICE, ONS, NCHS, or other definitions were applied (11). The PRIME study similarly demonstrated that prevalence varies when alternative post-COVID definitions are applied in positively and negatively tested adults (12).

### Sources of heterogeneity

Several factors likely explain the variability observed across studies. First, diagnostic definitions differed substantially. Some studies used WHO-defined PCC, whereas others used persistent symptoms lasting at least 12 weeks, broader 4-week post-acute symptom definitions, symptom-any definitions at fixed follow-up points, functional case definitions, or self-reported non-recovery. Our subgroup analysis showed that WHO-defined PCC produced lower pooled prevalence estimates than broader symptom-based definitions.

Second, follow-up duration varied across studies, ranging from approximately 3 months to several years. Prevalence estimates differed across follow-up categories, although heterogeneity remained high within each group. Follow-up duration is difficult to interpret in isolation because it is closely linked to study design, definition type, population severity, and symptom ascertainment method.

Third, hospitalization status and acute disease severity were important sources of variability. Hospitalized cohorts had higher pooled prevalence than non-hospitalized, community-based, or mixed cohorts. This is biologically and clinically plausible, as greater acute disease severity, oxygen requirement, intensive care, mechanical ventilation, and higher comorbidity burden have been associated with increased risk of persistent symptoms in post-discharge cohorts (13–16).

Fourth, age structure and pediatric inclusion may have contributed to heterogeneity. Pediatric cohorts, adult cohorts, and mixed-age studies may differ in symptom profiles, reporting patterns, functional impact, and recovery trajectories. Combining these populations in a global synthesis increases generalizability but reduces precision.

Fifth, symptom ascertainment varied widely. Many studies relied on self-reported questionnaires or telephone interviews, whereas others used clinical assessment, registry data, electronic health records, or structured instruments. Survey-based studies may capture a broader symptom burden but may overestimate clinically attributable Long COVID if baseline symptoms and alternative diagnoses are not accounted for. Conversely, registry- or code-based studies may underestimate prevalence because they capture only individuals who seek care and receive diagnostic coding (28).

Sixth, pandemic context likely affected prevalence estimates. The included literature spans different SARS-CoV-2 variant periods, pre-vaccination and vaccination eras, changes in testing availability, changes in healthcare access, and changing public awareness of Long COVID. Vaccination status and variant wave were not consistently reported across studies, but both are likely to influence the risk, severity, and detection of persistent post-COVID symptoms.

### Comparison with previous studies

Our pooled estimate is broadly consistent with the overall range reported in earlier systematic reviews, which have found substantial variability in Long COVID prevalence depending on the definition, population, and follow-up duration used (7–10). However, direct numerical comparison between reviews is difficult because inclusion criteria differ substantially. Some reviews focus on hospitalized cohorts, others on community-based samples, and others on specific definitions such as WHO-defined PCC.

The findings from this review align with previous evidence showing that stricter definitions generally yield lower prevalence estimates, whereas broader symptom-based definitions yield higher estimates. The present review extends this evidence by summarizing global data and explicitly emphasizing that the pooled estimate should be interpreted alongside prediction intervals and subgroup analyses rather than as a single global rate.

### Clinical and public health implications

Despite uncertainty around the exact prevalence, the findings indicate that Long COVID represents a substantial public health burden. Even if prevalence varies widely across settings, the large number of people infected with SARS-CoV-2 means that persistent post-COVID symptoms may generate long-term demand for healthcare services.

Healthcare systems should not rely solely on a single pooled prevalence figure for planning. Instead, service planning should use scenario-based estimates stratified by population type, disease severity, follow-up duration, and definition. Hospitalized patients, individuals with severe acute disease, patients requiring ICU care or advanced respiratory support, and those with key comorbidities may require more structured follow-up and targeted rehabilitation pathways.

The findings support the development of standardized post-COVID assessment pathways in primary care and specialist settings. These should include symptom screening, functional assessment, respiratory and cardiovascular evaluation when clinically indicated, cognitive and mental health assessment, and referral pathways for rehabilitation. Multidisciplinary care models may be particularly important because Long COVID is multisystemic and frequently includes fatigue, dyspnea, cognitive symptoms, sleep disturbances, psychological symptoms, and reduced functional capacity.

For surveillance, the key implication is that countries and health systems should report not only the label Long COVID or PCC but also the exact operational criteria used. Minimum reporting should include time since infection, symptom duration, symptom list or instrument, functional impairment requirement, exclusion of alternative diagnoses, hospitalization status, vaccination status, variant period when available, and whether baseline/control symptom data were used.

These considerations are especially important in low- and middle-income settings and in underrepresented regions, where standardized surveillance systems, access to post-COVID clinics, and diagnostic coding practices may differ substantially. Sparse data from some regions should not be interpreted as evidence of low burden; it may reflect limited ascertainment, reduced access to follow-up care, or under-coding.

### Strengths and limitations

This review has several strengths. First, it addresses an important global public health question using a systematic review and meta-analysis of observational studies. Second, it includes studies from multiple geographic regions and captures both hospitalized and non-hospitalized populations. Third, the analysis explicitly examined several major sources of variability, including hospitalization status, geographic region, operational case definition, and follow-up duration. Fourth, the revised analysis included prediction intervals, exploratory subgroup tests, leave-one-out sensitivity analysis, and a cohort-level sensitivity analysis including only one estimate per cohort or clinically distinct stratum.

Several limitations should also be acknowledged. The most important limitation is the extreme between-study heterogeneity. Although the pooled prevalence estimate provides a useful descriptive summary of the published literature, it should not be interpreted as a single precise global prevalence of Long COVID. The wide prediction interval indicates that prevalence estimates in future settings may vary substantially. This heterogeneity reflects differences in study design, population structure, hospitalization status, follow-up duration, symptom ascertainment, pandemic period, and operational case definition.

A second major limitation is the lack of uniform terminology and case definitions across the primary literature. Terms such as Long COVID, PCC, post-COVID syndrome, PASC, and persistent post-COVID symptoms were not used consistently across studies. Although some official frameworks provide specific definitions, primary epidemiological studies do not apply terminology and diagnostic criteria in a one-to-one manner. For this reason, we categorized estimates according to operational case definitions rather than terminology alone. Nevertheless, residual misclassification is possible because the exact implementation of symptom duration, functional impairment, exclusion of alternative diagnoses, and attribution to SARS-CoV-2 infection varied across studies.

Third, the inclusion criterion of follow-up at least 4 weeks after acute infection was intentionally broad. This allowed inclusion of early and broader Long COVID literature, where post-acute symptoms were often defined from 4 weeks onward. However, this threshold is broader than the WHO clinical case definition of PCC. Therefore, the overall pooled estimate should be interpreted as a synthesis of heterogeneous Long COVID-related operational definitions, whereas WHO-defined PCC was examined separately in subgroup analyses.

Fourth, many included studies relied on self-reported symptoms, questionnaires, or telephone interviews. These methods are valuable for capturing patient-reported symptom burden, but they may introduce recall bias, reporting bias, symptom awareness bias, and cultural differences in symptom reporting. In many studies, baseline pre-COVID symptom status was unavailable, and alternative diagnoses were not systematically excluded. This limits the ability to distinguish symptoms causally attributable to SARS-CoV-2 infection from background symptoms, comorbid conditions, or broader pandemic-related effects.

Fifth, follow-up duration varied substantially across studies, ranging from approximately 3 months to several years after acute infection. These time points may reflect different phases of recovery and should not be assumed to measure the same clinical phenomenon. Sixth, several studies contributed more than one prevalence estimate because they reported multiple follow-up time points, age groups, hospitalization strata, or operational definitions. We addressed this by conducting a cohort-level sensitivity analysis. The pooled prevalence remained essentially unchanged, but residual dependence cannot be fully excluded.

Seventh, adult and pediatric populations were included in the global synthesis. This increased the scope of the review but may have contributed to heterogeneity, because symptom profiles, reporting patterns, recovery trajectories, and functional consequences may differ between adults and children. Eighth, vaccination status, SARS-CoV-2 variant period, reinfection history, and acute treatment context were not consistently reported across studies. Ninth, regional representation was uneven. Finally, publication bias and small-study effects cannot be excluded. Funnel plot visualization and Egger's regression test did not show statistically significant asymmetry, but these methods have limited power and limited interpretability in prevalence meta-analyses with extreme heterogeneity.

The review protocol was not prospectively registered, which should be considered a methodological limitation. However, the eligibility criteria, data extraction procedures, and statistical analysis plan were defined before the quantitative synthesis was conducted.

### Future research

Future studies should prioritize standardized case definitions and transparent reporting of operational criteria. At minimum, studies should specify the time threshold, symptom duration requirement, symptom instrument, attribution rules, functional impairment requirement, exclusion of alternative diagnoses, and whether baseline or control-group symptom data were available.

Prospective longitudinal cohort studies with pre-infection or early post-infection baseline data are needed to distinguish incident post-COVID symptoms from background symptom prevalence. Test-negative control groups, population-based sampling, and linkage with clinical data would improve causal attribution and reduce overestimation. Future research should also report vaccination status, variant period, reinfection history, acute disease severity, hospitalization status, and treatment context. These variables are essential for understanding changes in Long COVID risk over time and for planning health-system responses.

Future reviews and meta-analyses should avoid relying solely on one pooled prevalence estimate. More informative approaches include stratified synthesis, prediction intervals, meta-regression when sufficient independent data are available, and scenario-based estimates for healthcare planning.

## Conclusion

This systematic review and meta-analysis provides an updated synthesis of observational evidence on the global prevalence of Long COVID/PCC. Across 22 studies contributing 27 prevalence estimates, the pooled prevalence was 30.8% (95% CI 26.8–35.0). However, heterogeneity was extreme, and the prediction interval was wide, indicating substantial variability across settings, populations, follow-up periods, and operational case definitions.

Therefore, the pooled prevalence should not be interpreted as a single precise global rate of Long COVID. Rather, it should be considered a broad descriptive summary of heterogeneous observational evidence. The main finding of this review is that reported Long COVID prevalence is highly dependent on how the condition is defined, when it is measured, which population is studied, and how symptoms are ascertained.

The results highlight a substantial long-term public health burden after SARS-CoV-2 infection, particularly among hospitalized and clinically vulnerable populations. At the same time, they underscore the need for standardized case definitions, harmonized follow-up protocols, transparent reporting of operational criteria, and improved surveillance systems.

For healthcare systems, these findings support the development of structured post-COVID assessment pathways, rehabilitation services, multidisciplinary care models, and targeted follow-up for high-risk groups. Future research should prioritize prospective longitudinal designs, standardized symptom and functional assessment, reporting of vaccination and variant context, and approaches that distinguish SARS-CoV-2-attributable symptoms from background symptom burden.

## Data Availability

The original contributions presented in the study are included in the article/Supplementary material, further inquiries can be directed to the corresponding author.
