# Supplementary material for: Global prevalence of post-COVID-19 condition (Long COVID): a systematic review and meta-analysis of observational studies
Source: Front Public Health. 2026 Jun 25;14:1839646. doi: 10.3389/fpubh.2026.1839646 (PMC13346190; doi:10.3389/fpubh.2026.1839646)
Supplement: Supplementary file 1 [file Table_1.docx]

**Supplementary Materials**

**Supplementary Table S1. Full database-specific search strategies**

| **Database** | **Search string** |
| --- | --- |
| PubMed/MEDLINE | (("COVID-19"[Mesh] OR "SARS-CoV-2"[Mesh] OR COVID-19[tiab] OR "coronavirus disease 2019"[tiab] OR SARS-CoV-2[tiab] OR "severe acute respiratory syndrome coronavirus 2"[tiab]) AND ("long COVID"[tiab] OR "Long-COVID"[tiab] OR "post-COVID condition"[tiab] OR "post-COVID-19 condition"[tiab] OR "post COVID condition"[tiab] OR "post COVID-19 condition"[tiab] OR "post-acute COVID"[tiab] OR "post-acute sequelae"[tiab] OR PASC[tiab] OR "persistent symptoms"[tiab] OR "post-COVID syndrome"[tiab] OR "post COVID syndrome"[tiab]) AND (prevalence[tiab] OR incidence[tiab] OR epidemiology[tiab] OR frequency[tiab] OR burden[tiab] OR cohort[tiab] OR "cross-sectional"[tiab] OR survey[tiab] OR registry[tiab] OR follow-up[tiab])) AND ("2020/01/01"[Date - Publication] : "2026/02/23"[Date - Publication]) |
| Scopus | TITLE-ABS-KEY(("COVID-19" OR "coronavirus disease 2019" OR "SARS-CoV-2" OR "severe acute respiratory syndrome coronavirus 2") AND ("long COVID" OR "Long-COVID" OR "post-COVID condition" OR "post-COVID-19 condition" OR "post COVID condition" OR "post COVID-19 condition" OR "post-acute COVID" OR "post-acute sequelae" OR PASC OR "persistent symptoms" OR "post-COVID syndrome" OR "post COVID syndrome") AND (prevalence OR incidence OR epidemiology OR frequency OR burden OR cohort OR "cross-sectional" OR survey OR registry OR follow-up)) AND PUBYEAR > 2019 AND PUBYEAR < 2027 |
| Web of Science Core Collection | TS=(("COVID-19" OR "coronavirus disease 2019" OR "SARS-CoV-2" OR "severe acute respiratory syndrome coronavirus 2") AND ("long COVID" OR "Long-COVID" OR "post-COVID condition" OR "post-COVID-19 condition" OR "post COVID condition" OR "post COVID-19 condition" OR "post-acute COVID" OR "post-acute sequelae" OR PASC OR "persistent symptoms" OR "post-COVID syndrome" OR "post COVID syndrome") AND (prevalence OR incidence OR epidemiology OR frequency OR burden OR cohort OR "cross-sectional" OR survey OR registry OR follow-up)); Timespan: 2020-2026 |
| WHO COVID-19 Global Literature Database | ("long COVID" OR "post-COVID-19 condition" OR "post COVID condition" OR "post-acute sequelae" OR PASC OR "persistent symptoms" OR "post-COVID syndrome") AND (prevalence OR incidence OR epidemiology OR frequency OR burden OR cohort OR survey OR registry OR follow-up) |

**Supplementary Table S2. Extracted prevalence estimates and inclusion in cohort-level sensitivity analysis**

| **Estimate ID** | **Cohort ID** | **Study / estimate** | **Country** | **Region** | **Population** | **Hospitalization status** | **Operational definition** | **Follow-up** | **N** | **Cases** | **Prevalence %** | **Included in cohort-level sensitivity** | **Reason** |
| --- | --- | --- | --- | --- | --- | --- | --- | --- | --- | --- | --- | --- | --- |
| E01 | C01 | Sørensen 2022 (EFTER-COVID) | Denmark | Europe | Adults/mixed (15+) | Mostly non-hospitalized | Broad/fixed follow-up symptom definition | 6–12 months | 61 002 | 18 057 | 29.6 | Yes | Retained as single eligible or clinically distinct estimate |
| E02 | C02 | Whitaker 2022 (REACT-2 rounds 3–5) | England | Europe | Adults | Mostly non-hospitalized | ≥12-week persistent symptoms | 3 months | 92 116 | 34 728 | 37.7 | Yes | Retained as single eligible or clinically distinct estimate |
| E03 | C02 | Whitaker 2022 (REACT-2 round 6) | England | Europe | Adults | Mostly non-hospitalized | ≥12-week persistent symptoms | 3 months | 14 562 | 3 145 | 21.6 | No | Repeated REACT-2 estimate; larger rounds 3–5 estimate retained |
| E04 | C03 | Peter 2022 (EPILOC) | Germany | Europe | Adults | Mostly non-hospitalized/mixed | Functional/study-specific | 8 months | 11 536 | 3 289 | 28.5 | Yes | Retained as single eligible or clinically distinct estimate |
| E05 | C04 | Förster 2022 | Germany | Europe | Adults | Mixed | ≥12-week persistent symptoms | 7 months | 1 459 | 720 | 49.3 | Yes | Retained as single eligible or clinically distinct estimate |
| E06 | C05 | Coste 2024 | France | Europe | Adults | Mixed | WHO-defined PCC | ≥3 months | 5 781 | 462 | 8.0 | Yes | Retained as single eligible or clinically distinct estimate |
| E07 | C06 | Montenegro 2022 | Spain | Europe | Adults | Mixed/primary care | ≥12-week persistent symptoms | 6 months | 579 | 83 | 14.3 | Yes | Retained as single eligible or clinically distinct estimate |
| E08 | C07 | Munblit 2021 (Moscow hospitals) | Russia | Europe | Adults | Hospitalized | Broad/fixed follow-up symptom definition | 6–8 months | 2 649 | 1 247 | 47.1 | Yes | Retained as single eligible or clinically distinct estimate |
| E09 | C08A | StopCOVID 2022 adults, 6 months | Russia | Europe | Adults | Hospitalized | WHO-defined PCC | 6 months | 1 013 | 507 | 50.0 | No | Repeated adult StopCOVID cohort; 12-month estimate retained |
| E10 | C08A | StopCOVID 2022 adults, 12 months | Russia | Europe | Adults | Hospitalized | WHO-defined PCC | 12 months | 1 013 | 344 | 34.0 | Yes | Retained as single eligible or clinically distinct estimate |
| E11 | C08B | StopCOVID 2022 children, 6 months | Russia | Europe | Children | Hospitalized | WHO-defined PCC | 6 months | 360 | 72 | 20.0 | No | Repeated pediatric StopCOVID cohort; 12-month estimate retained |
| E12 | C08B | StopCOVID 2022 children, 12 months | Russia | Europe | Children | Hospitalized | WHO-defined PCC | 12 months | 360 | 40 | 11.1 | Yes | Retained as single eligible or clinically distinct estimate |
| E13 | C09 | Hossain 2021 | Bangladesh | Asia | Adults | Mostly non-hospitalized | ≥12-week persistent symptoms | 3 months | 2 198 | 356 | 16.2 | Yes | Retained as single eligible or clinically distinct estimate |
| E14 | C10 | Khan/Hossain 2024 household survey | Bangladesh | Asia | Adults | Community | WHO-defined PCC | 6 months | 2 507 | 563 | 22.5 | Yes | Retained as single eligible or clinically distinct estimate |
| E15 | C11 | Iba 2024 (Japan BA.5) | Japan | Asia | Adults | Mostly non-hospitalized | WHO-defined PCC | 6 months | 8 392 | 990 | 11.8 | Yes | Retained as single eligible or clinically distinct estimate |
| E16 | C12 | Al-Adli 2024 (Qatar) | Qatar | Asia | Adults | Mostly non-hospitalized | WHO-defined PCC | ≥3 months | 368 | 159 | 43.2 | Yes | Retained as single eligible or clinically distinct estimate |
| E17 | C13 | India multicentre 2025 | India | Asia | Adults | Hospitalized | ≥12-week persistent symptoms | 12 months | 315 | 52 | 16.5 | Yes | Retained as single eligible or clinically distinct estimate |
| E18 | C14A | Jassat 2023 hospitalized | South Africa | Africa | Adults | Hospitalized | Broad/fixed follow-up symptom definition | 6 months | 2 626 | 1 227 | 46.7 | Yes | Retained as single eligible or clinically distinct estimate |
| E19 | C14B | Jassat 2023 non-hospitalized | South Africa | Africa | Adults | Non-hospitalized | Broad/fixed follow-up symptom definition | 6 months | 1 074 | 199 | 18.5 | Yes | Retained as single eligible or clinically distinct estimate |
| E20 | C15 | Tunisian cohort 2024 | Tunisia | Africa | Adults/mixed | Mixed | ≥12-week persistent symptoms | 3 months | 1 451 | 639 | 44.0 | Yes | Retained as single eligible or clinically distinct estimate |
| E21 | C16 | Brazil cohort 2024 | Brazil | South America | Adults/older adults | Mixed | Broad/fixed follow-up symptom definition | 12 months | 1 822 | 1 169 | 64.2 | Yes | Retained as single eligible or clinically distinct estimate |
| E22 | C17 | Alshahrani 2025 Saudi Arabia | Saudi Arabia | Asia | Adults | Mixed | WHO-defined PCC | 4 years | 816 | 238 | 29.2 | Yes | Retained as single eligible or clinically distinct estimate |
| E23 | C18 | Huang 2022 (Wuhan) 2-year | China | Asia | Adults | Hospitalized | Broad/fixed follow-up symptom definition | 24 months | 1 190 | 650 | 54.6 | Yes | Retained as single eligible or clinically distinct estimate |
| E24 | C19 | Yang 2022 (Wuhan) 1-year | China | Asia | Adults | Hospitalized | Broad/fixed follow-up symptom definition | 12 months | 1 864 | 806 | 43.2 | Yes | Retained as single eligible or clinically distinct estimate |
| E25 | C20A | Robineau 2022 hospitalized | France | Europe | Adults | Hospitalized | Broad/fixed follow-up symptom definition | 24 months | 360 | 215 | 59.7 | Yes | Retained as single eligible or clinically distinct estimate |
| E26 | C20B | Robineau 2022 non-hospitalized | France | Europe | Adults | Non-hospitalized | Broad/fixed follow-up symptom definition | 24 months | 308 | 208 | 67.5 | Yes | Retained as single eligible or clinically distinct estimate |
| E27 | C21 | Wuhan 3-year cohort 2024 | China | Asia | Adults | Hospitalized | Broad/fixed follow-up symptom definition | 36 months | 1 594 | 182 | 11.4 | Yes | Retained as single eligible or clinically distinct estimate |

***Supplementary Table S2. Characteristics of included studies and prevalence estimates. Some studies contributed more than one prevalence estimate because they reported different follow-up time points, age groups, hospitalization strata, or operational definitions. The column "Term used by authors" reports terminology used in the primary publication, whereas "Operational definition" reports the actual case definition applied for prevalence estimation. PCC, post-COVID-19 condition; WHO, World Health Organization.***

**Supplementary Table S3. Newcastle-Ottawa Scale-based risk-of-bias assessment**

| **Study / estimate** | **Selection** | **Comparability/representativeness** | **Outcome assessment** | **Follow-up/response completeness** | **Total score** | **Risk category** | **Main concerns** |
| --- | --- | --- | --- | --- | --- | --- | --- |
| Sørensen 2022 (EFTER-COVID) | 2 | 1 | 2 | 1 | 6 | Moderate | broad symptom definition |
| Whitaker 2022 (REACT-2 rounds 3–5) | 2 | 1 | 2 | 0 | 5 | Moderate–high | self-report/selection or follow-up bias |
| Whitaker 2022 (REACT-2 round 6) | 2 | 1 | 2 | 0 | 5 | Moderate–high | self-report/selection or follow-up bias |
| Peter 2022 (EPILOC) | 3 | 1 | 3 | 0 | 7 | Low–moderate | minor concerns |
| Förster 2022 | 2 | 1 | 2 | 1 | 6 | Moderate | minor concerns |
| Coste 2024 | 2 | 1 | 2 | 0 | 5 | Moderate–high | self-report/selection or follow-up bias |
| Montenegro 2022 | 2 | 1 | 2 | 0 | 5 | Moderate–high | self-report/selection or follow-up bias |
| Munblit 2021 (Moscow hospitals) | 2 | 1 | 2 | 0 | 5 | Moderate–high | self-report/selection or follow-up bias; broad symptom definition; limited generalizability to community cases |
| StopCOVID 2022 adults, 6 months | 2 | 1 | 2 | 0 | 5 | Moderate–high | self-report/selection or follow-up bias; limited generalizability to community cases |
| StopCOVID 2022 adults, 12 months | 2 | 1 | 2 | 0 | 5 | Moderate–high | self-report/selection or follow-up bias; limited generalizability to community cases |
| StopCOVID 2022 children, 6 months | 2 | 1 | 2 | 0 | 5 | Moderate–high | self-report/selection or follow-up bias; limited generalizability to community cases |
| StopCOVID 2022 children, 12 months | 2 | 1 | 2 | 0 | 5 | Moderate–high | self-report/selection or follow-up bias; limited generalizability to community cases |
| Hossain 2021 | 2 | 1 | 2 | 0 | 5 | Moderate–high | self-report/selection or follow-up bias |
| Khan/Hossain 2024 household survey | 2 | 1 | 2 | 1 | 6 | Moderate | minor concerns |
| Iba 2024 (Japan BA.5) | 3 | 1 | 3 | 0 | 7 | Low–moderate | minor concerns |
| Al-Adli 2024 (Qatar) | 2 | 1 | 2 | 0 | 5 | Moderate–high | self-report/selection or follow-up bias |
| India multicentre 2025 | 2 | 1 | 2 | 0 | 5 | Moderate–high | self-report/selection or follow-up bias; limited generalizability to community cases |
| Jassat 2023 hospitalized | 2 | 1 | 2 | 0 | 5 | Moderate–high | self-report/selection or follow-up bias; broad symptom definition; limited generalizability to community cases |
| Jassat 2023 non-hospitalized | 2 | 1 | 2 | 0 | 5 | Moderate–high | self-report/selection or follow-up bias; broad symptom definition |
| Tunisian cohort 2024 | 2 | 1 | 2 | 0 | 5 | Moderate–high | self-report/selection or follow-up bias |
| Brazil cohort 2024 | 2 | 1 | 2 | 1 | 6 | Moderate | broad symptom definition |
| Alshahrani 2025 Saudi Arabia | 2 | 1 | 2 | 1 | 6 | Moderate | minor concerns |
| Huang 2022 (Wuhan) 2-year | 2 | 1 | 2 | 0 | 5 | Moderate–high | self-report/selection or follow-up bias; broad symptom definition; limited generalizability to community cases |
| Yang 2022 (Wuhan) 1-year | 2 | 1 | 2 | 0 | 5 | Moderate–high | self-report/selection or follow-up bias; broad symptom definition; limited generalizability to community cases |
| Robineau 2022 hospitalized | 2 | 1 | 2 | 0 | 5 | Moderate–high | self-report/selection or follow-up bias; broad symptom definition; limited generalizability to community cases |
| Robineau 2022 non-hospitalized | 2 | 1 | 2 | 0 | 5 | Moderate–high | self-report/selection or follow-up bias; broad symptom definition |
| Wuhan 3-year cohort 2024 | 2 | 1 | 2 | 0 | 5 | Moderate–high | self-report/selection or follow-up bias; broad symptom definition; limited generalizability to community cases |

***Supplementary Table S3. Newcastle-Ottawa Scale-based risk-of-bias assessment. Domains included selection, comparability/representativeness, outcome assessment, follow-up or response completeness, and main concerns.***

**Supplementary Table S4. Leave-one-out sensitivity analysis**

| **Excluded estimate ID** | **Excluded estimate** | **k retained** | **Pooled prevalence %** | **95% CI** | **I² %** | **Interpretation** |
| --- | --- | --- | --- | --- | --- | --- |
| E01 | Sørensen 2022 (EFTER-COVID) | 26 | 30.8 | 25.8–36.3 | 99.7 | Stable; no single estimate dominated |
| E02 | Whitaker 2022 (REACT-2 rounds 3–5) | 26 | 30.5 | 25.7–35.7 | 99.6 | Stable; no single estimate dominated |
| E03 | Whitaker 2022 (REACT-2 round 6) | 26 | 31.2 | 27.2–35.5 | 99.6 | Stable; no single estimate dominated |
| E04 | Peter 2022 (EPILOC) | 26 | 30.8 | 26.7–35.4 | 99.7 | Stable; no single estimate dominated |
| E05 | Förster 2022 | 26 | 30.1 | 26.2–34.4 | 99.7 | Stable; no single estimate dominated |
| E06 | Coste 2024 | 26 | 32.2 | 28.4–36.2 | 99.6 | Stable; no single estimate dominated |
| E07 | Montenegro 2022 | 26 | 31.5 | 27.5–35.9 | 99.7 | Stable; no single estimate dominated |
| E08 | Munblit 2021 (Moscow hospitals) | 26 | 30.2 | 26.2–34.5 | 99.7 | Stable; no single estimate dominated |
| E09 | StopCOVID 2022 adults, 6 months | 26 | 30.1 | 26.2–34.4 | 99.7 | Stable; no single estimate dominated |
| E10 | StopCOVID 2022 adults, 12 months | 26 | 30.6 | 26.6–35.0 | 99.7 | Stable; no single estimate dominated |
| E11 | StopCOVID 2022 children, 6 months | 26 | 31.2 | 27.2–35.6 | 99.7 | Stable; no single estimate dominated |
| E12 | StopCOVID 2022 children, 12 months | 26 | 31.7 | 27.7–36.1 | 99.7 | Stable; no single estimate dominated |
| E13 | Hossain 2021 | 26 | 31.5 | 27.5–35.8 | 99.7 | Stable; no single estimate dominated |
| E14 | Khan/Hossain 2024 household survey | 26 | 31.1 | 27.1–35.5 | 99.7 | Stable; no single estimate dominated |
| E15 | Iba 2024 (Japan BA.5) | 26 | 31.8 | 28.0–35.8 | 99.6 | Stable; no single estimate dominated |
| E16 | Al-Adli 2024 (Qatar) | 26 | 30.3 | 26.4–34.6 | 99.7 | Stable; no single estimate dominated |
| E17 | India multicentre 2025 | 26 | 31.4 | 27.4–35.7 | 99.7 | Stable; no single estimate dominated |
| E18 | Jassat 2023 hospitalized | 26 | 30.2 | 26.2–34.5 | 99.7 | Stable; no single estimate dominated |
| E19 | Jassat 2023 non-hospitalized | 26 | 31.3 | 27.3–35.7 | 99.7 | Stable; no single estimate dominated |
| E20 | Tunisian cohort 2024 | 26 | 30.3 | 26.3–34.6 | 99.7 | Stable; no single estimate dominated |
| E21 | Brazil cohort 2024 | 26 | 29.6 | 25.9–33.7 | 99.7 | Stable; no single estimate dominated |
| E22 | Alshahrani 2025 Saudi Arabia | 26 | 30.8 | 26.8–35.2 | 99.7 | Stable; no single estimate dominated |
| E23 | Huang 2022 (Wuhan) 2-year | 26 | 29.9 | 26.0–34.2 | 99.7 | Stable; no single estimate dominated |
| E24 | Yang 2022 (Wuhan) 1-year | 26 | 30.3 | 26.3–34.6 | 99.7 | Stable; no single estimate dominated |
| E25 | Robineau 2022 hospitalized | 26 | 29.8 | 25.9–34.0 | 99.7 | Stable; no single estimate dominated |
| E26 | Robineau 2022 non-hospitalized | 26 | 29.6 | 25.7–33.7 | 99.7 | Stable; no single estimate dominated |
| E27 | Wuhan 3-year cohort 2024 | 26 | 31.8 | 27.8–36.1 | 99.7 | Stable; no single estimate dominated |

***Supplementary Table S4. Leave-one-out sensitivity analysis. The pooled prevalence remained between 29.2% and 32.1% after sequential exclusion of individual estimates, indicating that no single estimate disproportionately influenced the overall result***
